# Dogs as Sentinels for Human Infection with Japanese Encephalitis Virus

**DOI:** 10.3201/eid1607.091757

**Published:** 2010-07

**Authors:** Hiroshi Shimoda, Yoshito Ohno, Masami Mochizuki, Hiroyuki Iwata, Masaru Okuda, Ken Maeda

**Affiliations:** Author affiliations: Yamaguchi University, Yoshida, Yamaguchi, Japan (H. Shimoda, Y. Ohno, H. Iwata, M. Okuda, K. Maeda);; Kyoritsu Seiyaku Corporation, Kudankita, Chiyoda-ku, Tokyo, Japan (M. Mochizuki)

**Keywords:** Sentinel surveillance, encephalitis, Japanese, dogs, viruses, vector-borne infections, dispatch

## Abstract

Because serosurveys of Japanese encephalitis virus (JEV) among wild animals and pigs may not accurately reflect risk for humans in urban/residential areas, we examined seroprevalence among dogs and cats. We found that JEV-infected mosquitoes have spread throughout Japan and that dogs, but not cats, might be good sentinels for monitoring JEV infection in urban/residential areas.

Japanese encephalitis virus (JEV), a common cause of serious acute encephalitis in humans, is primarily transmitted by *Culex tritaeniorhynchus* mosquitoes and is widely endemic to Southeast Asia and the Western Pacific region (*1*). Annual incidence of Japanese encephalitis (JE) is ≈50,000 cases,with 10,000 deaths (*2*). In Japan during the 1950s, several thousand JE cases occurred each year. However, as a result of a JEV vaccination program, isolation of pig farms, and reduction of mosquitoes, the number of JE cases in Japan has decreased markedly, to <10 cases per year since 1992 (*3*). In 2005, the strong recommendation for JE vaccination was halted because of a severe vaccine-associated side effect in 1 person; however, since 2009, a newly developed JE vaccine has been available and vaccination has been resumed.

Annual serosurveys for JEV antibodies in pigs, the main amplifiers of JEV, tend to show high seropositivity in western Japan (*3*). Our previous study of JEV in wild animals in the Kinki district also showed high seroprevalence: 83% among wild boars and 59% among raccoons (*4*). These data indicate that JEV remains endemic to Japan.

However, serosurveys of wild animals and pigs may not accurately reflect risk for humans because these animals remain separate from human populations and thus may not indicate the prevalence of JEV in urban/residential areas of Japan. Therefore, additional monitoring of the risk for JEV infection in humans in these areas, in addition to annual surveillance of pigs, is needed. To determine seroprevalence in family-owned dogs and cats, which share living space with humans, we conducted serosurveys of JEV in these species.

## The Study

First, to examine whether dogs and cats were infected with JEV, we analyzed serum samples from 100 dogs and 292 cats in Yamaguchi Prefecture, which is in the western part of Honshu, Japan. Dog samples were collected during 2006–2007, and cat samples were collected during 1997–1999 and 2004–2005. An 80% plaque-reduction neutralizing test using virus JEV/sw/Chiba/88/2002 was performed as described (*4*). Virus JEV/sw/Chiba/88/2002 is genetically classified as genotype I (*5*). To analyze the results statistically, we performed χ^2^ and Fisher exact probability tests. The significance level was p<0.05.

Results showed that 17% of dogs and 1% of cats were seropositive for JEV; thus, seropositivity was ≈10-fold higher among dogs than among cats (Table 1). In addition, outdoor-only dogs (38%) were 3.7-fold more likely to be seropositive than were indoor-only dogs (10%) (p<0.05). Antibody prevalence did not differ significantly between male (14%) and female (20%) dogs (p>0.05; data not shown).

**Table 1:** Seroprevalence of Japanese encephalitis virus among dogs (2006–2007) and cats (1997–2005), Yamaguchi, Japan*

Animals	No. examined	No. (%) positive
Dogs		
Indoor	58	6 (10)
Outdoor	21	8 (38)
Both or unknown	21	3 (14)
Total	100	17 (17)
Cats	292	3 (1)

*Housing information obtained by questionnaire.

Next, serum samples from 652 dogs in every district in Japan during 2006–2007 were examined for seroprevalence of JEV. The results showed that 25% of dogs had virus-neutralizing antibodies against JEV. In northern Japan, 0% and 9% of dogs from the Hokkaido and Tohoku districts, respectively, were seropositive; these levels were significantly lower than those for other districts (p<0.05). In contrast, in southern Japan, 61% and 47% of dogs in the Shikoku and Kyushu districts, respectively, were seropositive; these levels were significantly higher than those for other districts (p<0.05). Seropositivity to JEV in the Kanto (17%), Chubu (18%), Kinki (23%), and Chugoku (26%) districts showed no significant differences (p>0.05) (Figure). In addition, 45% of outdoor-only dogs and 8% of indoor-only dogs were seropositive for JEV, thus confirming that outdoor-only dogs were 5.5-fold more likely than indoor-only dogs to be seropositive (p<0.05) (Table 2). Regarding the areas of residence, 21% of dogs in urban/residential areas and 43% of dogs in rural areas were seropositive; the results for rural areas were significantly higher than those for urban/residential areas (Table 2). No significant correlation was found between ages of dogs and JEV seropositivity (data not shown).

**Figure:**
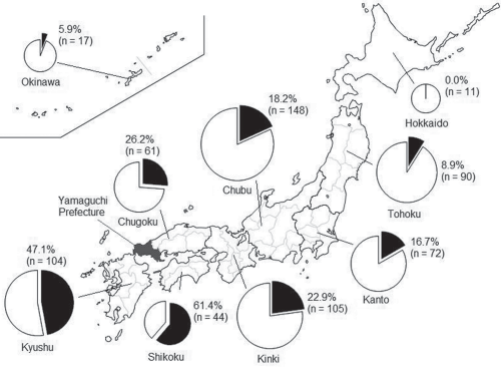
Seropositivity for Japanese encephalitis virus among dogs in 9 districts of Japan, 2006–2007. Numbers in parenthesis indicate number of dogs tested. The size of each circle indicates the number of samples. Black pie chart segments indicate the proportion of seropositive dogs; white segments indicate proportion of seronegative dogs.

**Table 2:** Seroprevalence of Japanese encephalitis virus among dogs throughout Japan, 2006–2007*

Location	No. examined	No. (%) positive
Where dog stays		
Indoors	222	18 (8)
Outdoors	234	105 (45)
Both or unknown	196	41 (21)
Type of area		
Urban/residential	405	86 (21)
Rural	152	65 (43)
Unknown	95	13 (14)

*Housing information obtained by questionnaire.

## Discussion and Conclusions

Our findings of significantly higher JEV seropositivity among dogs than cats are similar to those found in a 1954–1955 study in which 55% of dogs and 10% of cats were seropositive for JEV (*6*). Studies of another mosquito-vectored virus found 26% of dogs and 9% of cats in Louisiana and 5% of dogs and no cats in New York to be seropositive for West Nile virus (WNV) (*7**,**8*). Previous reports on host feeding patterns of JEV and WNV vectors showed that although *Culex* spp. mosquitoes feed on various mammals, including dogs, cats, and humans, they tend to feed more on dogs than on cats or humans (*9**,**10*). These reports are consistent with our finding that seropositivity was higher among dogs than among cats and humans and indicate that some JEV vectors do occasionally feed on humans.

Our nationwide serosurvey indicated that JEV prevalence was significantly lower in the Hokkaido and Tohoku districts and significantly higher in the Kyushu and Shikoku districts (Figure). Annual serosurveys of pigs have also shown that JEV seropositivity rates are higher for pigs in western than in northern Japan. In addition, during 2005–2007 in Japan, 24 JE cases in humans were reported, most of which occurred in western Japan (*11*). This finding is consistent with our data, suggesting that serosurveys in dogs accurately reflect JEV infection risk for humans in Japan.

Our finding that 45% of outdoor-only dogs were seropositive for JEV is similar to the finding of a previous study, conducted during a WNV epidemic among humans, that 69% of outdoor-only dogs were seropositive for WNV (*7*). A serosurvey in the Kanto district of Tokyo during 1954–1955 showed that 49% of stray dogs were seropositive for JEV (*6*). Results of these studies are similar to our results, indicating that risk for JEV infection remains high in Japan, particularly in the western part. In addition, confirmation of seropositivity among indoor-only dogs (8%) indicates that JEV-infected mosquitoes may enter houses; thus, infants and elderly persons, who tend to go outside less frequently, might also be at risk for JEV infection.

That seropositivity in rural areas (43%) was significantly higher than that in urban/residential areas (21%) suggests that pig farms and rice paddies in rural areas are associated with JEV. However, the relatively high seropositivity in urban/residential areas suggests that JEV infection risk for humans remains high, even in areas with few pig farms and rice paddies. Because pigs are housed away from humans, serosurveys of pigs in urban/residential areas are limited. Therefore, dogs, which are found in all areas of Japan, may be better sentinels for JEV infection in these areas. However, information such as whether dogs become viremic after JEV infection or how long anti-JEV antibodies last in them remains unclear.

In conclusion, using dogs as sentinels indicated that the risk for human infection with JEV remains high, even in urban/residential areas. Therefore, to assess the continuing risk for JEV infection in humans in urban/residential areas of Japan, we recommend JEV surveillance among pigs every year and surveillance among dogs every several years.
